# Comparative Evaluation of Three Different Demineralisation Protocols on the Physicochemical Properties and Biocompatibility of Decellularised Extracellular Matrix for Bone Tissue Engineering Applications

**DOI:** 10.7759/cureus.64813

**Published:** 2024-07-18

**Authors:** Parkavi Arumugam, G Kaarthikeyan, Rajalakshmanan Eswaramoorthy

**Affiliations:** 1 Periodontics, Saveetha Dental College and Hospitals, Saveetha Institute of Medical and Technical Sciences, Saveetha University, Chennai, IND; 2 Biochemistry, Center of Molecular Medicine and Diagnostics (COMManD), Saveetha Dental College and Hospitals, Saveetha Institute of Medical and Technical Sciences, Saveetha University, Chennai, IND

**Keywords:** bone, decellularised extracellular matrix, extracellular matrix, bioink, tissue engineering, bioprinting, regeneration, periodontitis

## Abstract

Background

With three-dimensional (3D) bioprinting emerging as the ultimate pinnacle of personalised treatment for achieving predictable regenerative outcomes, the search for tissue-specific bioinks is on. Decellularised extracellular matrix (DECM), which provides the inherent biomimetic cues, has gained considerable attention. The objective of the present study was to compare the efficacy of three different demineralisation protocols to obtain DECM for bone tissue engineering applications.

Methodology

Goat femurs were treated using three demineralisation protocols to obtain DECM. Group A was treated with demineralisation solution at 40 rpm for 14 days, Group B with freeze-thaw cycles and 0.05M hydrochloric acid (HCl) and 2.4 mM ethylenediamine tetra-acetic acid (EDTA) at 40 rpm for 60 days, and Group C with 0.1M HCl at 40 rpm for three days. After washing, neutralization, 0.05% trypsin-EDTA treatment for 24 hours, and lyophilisation, DECM was obtained. Assessments included scanning electron microscope (SEM) analysis, energy dispersive X-ray (EDX) analysis, hematoxylin and eosin (H&E) staining, and biocompatibility analysis.

Results

On comparative analysis, the protocol followed by Group C revealed good surface properties with patent and well interconnected pores with an average pore size of 218.87µm. Group C also revealed carbon and oxygen as predominant components with trace amounts of calcium, proving adequate demineralisation. Group C further revealed optimal demineralisation and decellularisation under histological analysis while maintaining biocompatibility. DECM obtained in Group C should be further processed for bioprinting applications.

Conclusion

The three protocols explored in this study hold potential, with Group C's protocol demonstrating the most promise for DECM-based bioink applications. Further research is needed to evaluate the suitability of the obtained DECM for preparing tissue-specific bioinks for 3D bioprinting.

## Introduction

Bone defects are one of the most common findings of the periodontal disease process [1]. Various surgical regenerative materials and techniques like bone grafting, guided tissue regeneration, and enamel matrix proteins are currently being used in the treatment of infrabony defects [2,3]. Recent advances in tissue engineering have led to the incorporation of three-dimensional (3D) bioprinting techniques for bone grafting. 3D bioprinting is a revolutionary additive manufacturing technology that enables printing three-dimensional osseous scaffolds using bioinks. Bioinks are a formulation of cells and biomaterials with physiologically active components providing biologic cues for tissue regeneration, that can be applied using automated biofabrication technologies [4]. Selecting the appropriate bioink is the most crucial step in the bioprinting process. The bioinks should possess the property of shear thinning which allows its extrusion through the nozzle tips during printing while maintaining shape fidelity post-printing [5]. Various natural, synthetic, and composite hydrogels based on biomaterials like collagen, gelatin, alginate, chitosan, agar, chondroitin sulfate, polycaprolactone, polyethylene glycol, hydrogels, and decellularised extracellular matrix (DECM) are being explored as potential bioinks [6]. 

Crosslinking of the bioink components is a primary requisite to obtain a printable bioink. Physical and chemical crosslinking are the two most commonly used methods [7]. Physical crosslinkers are associated with problems related to the speed of crosslinking while chemical crosslinkers are associated with poor biocompatibility [8]. Various chemical modifications of the bioinks have been explored to overcome this challenge. Recent studies have shown that chemically modified extracellular matrix (ECM)-based hydrogels exhibit superior biofunctionality and mechanical tunability [9,10]. DECM bioinks provide the advantage of enriching the regenerative potential of the biomaterials. Its rich composition of tissue-specific bioactive proteins and factors have been postulated to play a crucial role in cell differentiation and proliferation by regulating bioactive molecules, receptor levels, and microenvironmental pH. Moreover, its compositional and structural similarity creates a bioink that is more conducive for bioprinting through extrusion while requiring minimal external crosslinking elements to be added to the bioink. Hence, DECM bioinks provide a unique tissue specific composition with increased bio-functionality, that is completely natural without additional external crosslinkers, which would ultimately increase the biocompatibility and regenerative outcomes. 

DECM-based bioinks are natural alternative biomaterials of interest that mimic the native ECM which influences cell attachment, survival, and differentiation [11]. They are tissue matrices that are devoid of cellular and immunogenic content while retaining the structure of ECM with components like collagen, elastin, laminin, and glycosaminoglycans. This provides the required structural and mechanical integrity along with biological cues for cell seeding. 

DECM can be classified based on the source of the tissue into autogenous DECM, allogenous DECM, and xenogenous DECM [12]. Autogenous DECM though ideal is associated with morbidity and limited tissue availability [13]. Allogenic and xenogenic DECM are the alternative attractive options. They can also be categorised as organ/tissue-derived DECM and cell-derived DECM [12]. Organ/tissue-derived DECM possess the native 3D architecture with sophisticated memory cues that play a role in site-specific cell attachment and differentiation [14]. Cell-derived DECM are easier to harvest with reduced risk of pathogen transfer. They are more adaptable and modifiable for application in conjunction with other biomaterials. 

Decellularisation of ECM is a tissue-specific process that is achieved with physical, chemical, enzymatic, and combination protocols. Physical methods consist of a freeze-thaw cycle, perfusion, immersion, and agitation while chemical methods consist of treatment with ionic-nonionic detergents, acids-bases, and hypotonic-hypertonic solutions [12]. Enzymatic methods include treatment with nucleases, and trypsin to remove all the cellular and immunogenic components [12].To date, no gold standard method of decellularisation has been established. Moreover, obtaining DECM from bone tissue requires a critical aspect of demineralisation of the mineral content of the bone along with decellularisation of the ECM. This study aimed to compare and analyse the effect of three different demineralisation protocols along with the physiochemical properties and biocompatibility of the DECM. The DECM thus derived from the bone tissue would be further processed for bioink formulations. 

## Materials and methods

The study was conducted at the Department of Biomaterials, Saveetha Dental College, Chennai, India. Approval for the conduction of the study was obtained from the Scientific Review Board of Saveetha Dental College (SRB/SDC/PhD/PERIO-2264/23/084). All the chemicals and materials used in the study were of analytical grade and were procured from Sigma Aldrich, Merck Group, Darmstadt, Germany. MG-63 cells for the biocompatibility analysis were obtained from the National Centre for Cell Science, Pune, India. The growth media and supplements were procured from HiMedia Labs Pvt Ltd., Thane, India. 

Bone procurement and preparation 

Fresh goat femur bone was harvested from a 12- to 24-month-old goat at a local slaughterhouse. The bones were cut into cortical and cancellous segments and the superficially attached muscles and tendons were removed. The bones were processed in three phases for removal of the immunogenic components. Phase I consisted of demineralisation followed by Phase II of decellularisation which was completed with Phase III of thorough washing in distilled water for removal of any residues. In Phase I the bones were divided into three groups. Each group was treated with a different demineralisation protocol with different acid concentrations, agitation speed, and treatment duration. All the groups were then treated with similar Phase II and III protocols. The samples were stored at -80°C in the interim period between different processing stages. 

Phase I: Demineralisation Protocol

Group A: A fresh batch of 1000 ml of demineralisation solution was prepared with a composition of 323.4 mg of 0.1 M calcium chloride, 450 μl of 0.01 M acetic acid, and 300 mg of 0.1 M potassium dihydrogen phosphate. The cut bone pieces were immersed in the demineralisation solution at room temperature and allowed for continuous gentle agitation using orbital shaker at 40 rpm for 14 days. The demineralising solution was changed at periodic intervals of every two days.

Group B: The bone segments were first physically treated with three repetitive cycles of one hour of freezing at -80°C followed by one hour of thawing in distilled water at room temperature. They were then immersed and allowed for continuous gentle agitation using orbital shaker in a solution of 0.05M hydrochloric acid (HCl) and 2.4 mM ethylenediaminetetraacetic acid (EDTA) at 40 rpm for six days at room temperature.

Group C: This group was treated with immersion and agitation using orbital shaker in 1 M HCl at 40 rpm at room temperature for three days. 

Phase II: Decellularisation Protocol

Post demineralisation, the softened bones were placed in distilled water till the bones were completely immersed. They were thoroughly washed by continuous gentle agitation in distilled water for one day. The bones were then cut into small pieces measuring 1cm x 1cm x 0.5cm. They were then neutralised with immersion and continuous gentle agitation in 5% sodium sulfate solution for 24 hours at room temperature. Following neutralisation, the bones were treated for decellularisation with immersion and gentle continuous agitation in 0.05% trypsin-EDTA solution at room temperature for 24 hours. 

Phase III: Post-treatment Washing

The bone pieces were immersed in distilled water till the water was double the volume of the bone pieces. They were then thoroughly washed by continuous gentle agitation in distilled water for two days, allowing for removal of any residual acid. The water was changed daily for the washing. The resultant demineralised and decellularised bone samples, now referred to as DECM, were then freeze-dried at -80°C for one day and lyophilised at -90°C for two days. The lyophilised samples were stored at 4°C till further testing.

SEM and EDX analysis

The samples were analysed with field emission scanning electron microscope (SEM) JEOL JSM IT800 (Peabody, MA, USA). The tissue samples were platinum sputter coated for 30 seconds and viewed under the microscope at 100 micrometers. Energy dispersive X-ray (EDX) analysis was done to chemically characterise the samples. 

Histopathologic analysis

The lyophilized specimens underwent slicing into thin sections and were subsequently immersed in 10% neutral buffered formalin for fixation. These samples exhibited a consistency ranging from soft to firm, with approximate dimensions of 0.6 x 0.5 x 1 cm. Following grossing and labeling, the specimens underwent manual dehydration and were cleared using various clearing agents. Subsequently, they were embedded in paraffin wax and sectioned. Each sample yielded three sections measuring 4 µm in thickness. These sections were then subjected to staining using a routine and standardized hematoxylin & eosin (H & E) staining protocol.

Biocompatibility analysis

To assess the biocompatibility of the DECM samples, 3-[4,5-dimethylthiazol-2-yl]-2,5 diphenyl tetrazolium bromide (MTT) assay was performed by analysing the cell viability. 1 mg/ml of demineralised samples were immersed in minimum essential medium Eagle - alpha modification (α-MEM) supplemented with 1% antibiotic solution for 24 hrs. The media was collected after 24 hrs in the syringe, filtered, and used as treatment media. The biocompatibility of the prepared samples was tested against osteoblast (MG63) cell lines. MG63 cells were cultured at the density of 0.05 x 10^6^ cells per well and treated with the treatment media. The MTT assay was performed after 24 hours of treatment. The spent media was removed and 10 µl/100 ml of MTT reagent (5mg/ml of stock) was added. The plates were incubated for four hours at 37°C. The MTT reagent was exchanged with dimethyl sulfoxide (DMSO) to dissolve the formed formazan crystals. The absorbance was measured at 570 nm using an enzyme-linked immunoassay (ELISA) plate reader. The tests were performed in triplicates and the percentage of cell viability was calculated.

Statistical analysis

IBM SPSS Statistics for Windows, Version 23.0 (Released 2015; IBM Corp., Armonk, NY, USA), was used to analyze the data. The impact of the three different demineralisation protocols on the biocompatibility of the DECM samples were compared using a one-way ANOVA with post-hoc Tukey's honestly significant difference (HSD), with a p-value of 0.05 designated as statistically significant. 

## Results

SEM analysis

**Figure 1:**
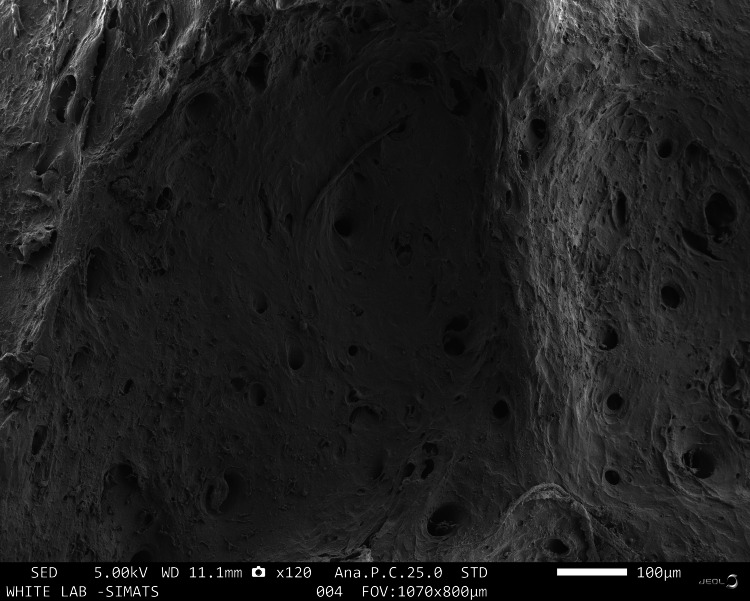
SEM image of DECM sample of Group A SEM: scanning electron microscope, DECM: decellularised extracellular matrix

**Figure 2:**
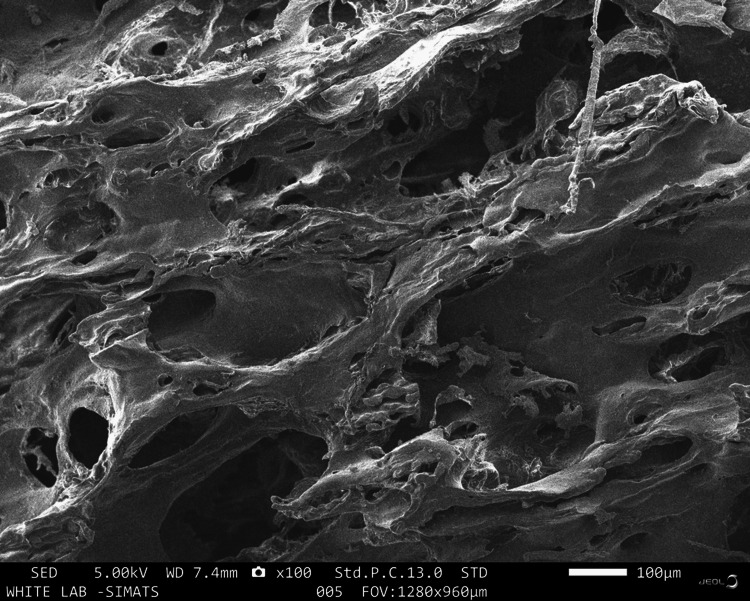
SEM image of DECM sample of Group B SEM: scanning electron microscope, DECM: decellularised extracellular matrix

**Figure 3:**
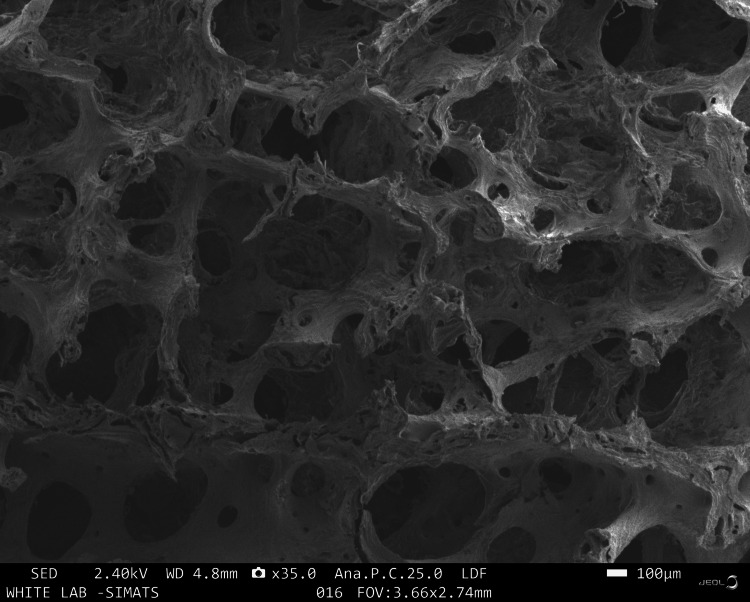
SEM image of DECM sample of Group C SEM: scanning electron microscope, DECM: decellularised extracellular matrix

Under a scanning electron microscope, all three samples appeared to be adequately decellularised as observed in Figures 1-3. Group A revealed a less trabecular structure with porosities ranging from 14.77µm to 51.93µm. The average size of the porosity was 30.31µm and some of the pores were partially covered with fibrillar structures. Glomerular agglomerates were also noted on the surface of the samples which may be suggestive of debris. Group B revealed a rough and trabecular surface morphology with widely varying pore sizes. The porosities ranged from 7.105µm to 73.08µm with an average pore size of 34.62µm. The porosities were well interconnected and the 3D architecture of the scaffold was well appreciable. Group C revealed the most well-defined and appreciable trabecular architecture of the scaffold. The pore size of Group C was the greatest of all the samples which may be attributed to the better demineralisation protocol along with the inherent bone anatomy of the sample. The porosities ranged from 62.18µm to 466.3µm with an average pore size of 218.87µm. The pores were also well interconnected with well-established patency of the pores. The lamellar architecture of the ECM was the most distinctly appreciable in Group C followed by Group B. Similar results were noted with other studies that analysed the surface morphology of different tissue specimens and revealed samples that were devoid of any cellular material with the presence of well-appreciable lamellar collagen structures [15]. 

EDX analysis

**Figure 4:**
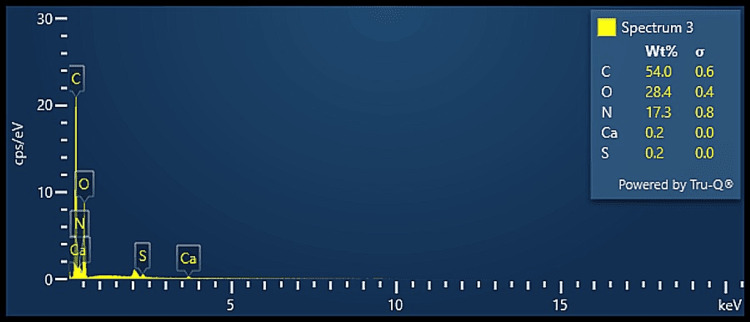
EDX analysis of DECM sample of Group A EDX: energy dispersive X-ray, DECM: decellularised extracellular matrix

**Figure 5:**
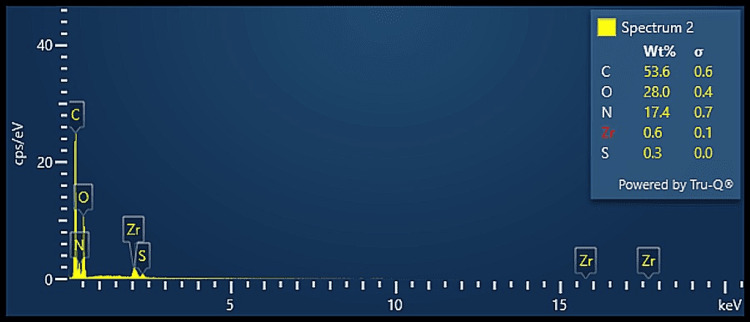
EDX analysis of DECM sample of Group B EDX: energy dispersive X-ray, DECM: decellularised extracellular matrix

**Figure 6:**
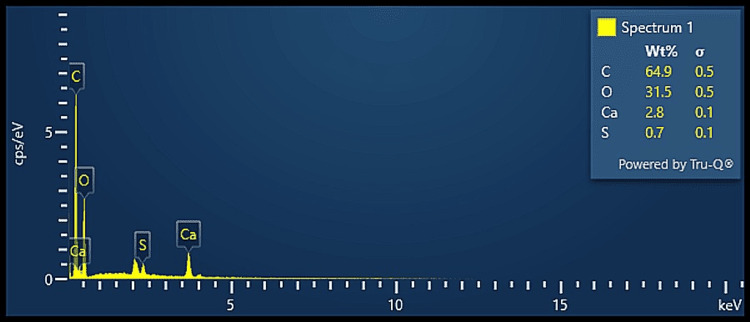
EDX analysis of DECM sample of Group C EDX: energy dispersive X-ray, DECM: decellularised extracellular matrix

The elemental composition analysis using EDX, as seen in Figures 4-6, revealed that all three groups revealed the presence of carbon and oxygen as the predominant components, which may suggest the presence of ECM collagen. Group A also revealed the presence of nitrogen along with trace amounts of calcium and sulfur while Group B revealed the presence of nitrogen and trace amounts of zirconia and sulfur. Group C revealed the presence of trace amounts of calcium and sulfur. As all the groups revealed very trace amounts of calcium, it proves that the bone samples were sufficiently demineralised along with the achievement of adequate decellularisation. The results are in accordance with a recent study that analysed the decellularized sheets of mineralized collagen fibrils obtained from a common digital extensor of the steer [16].

Histopathologic analysis

**Figure 7:**
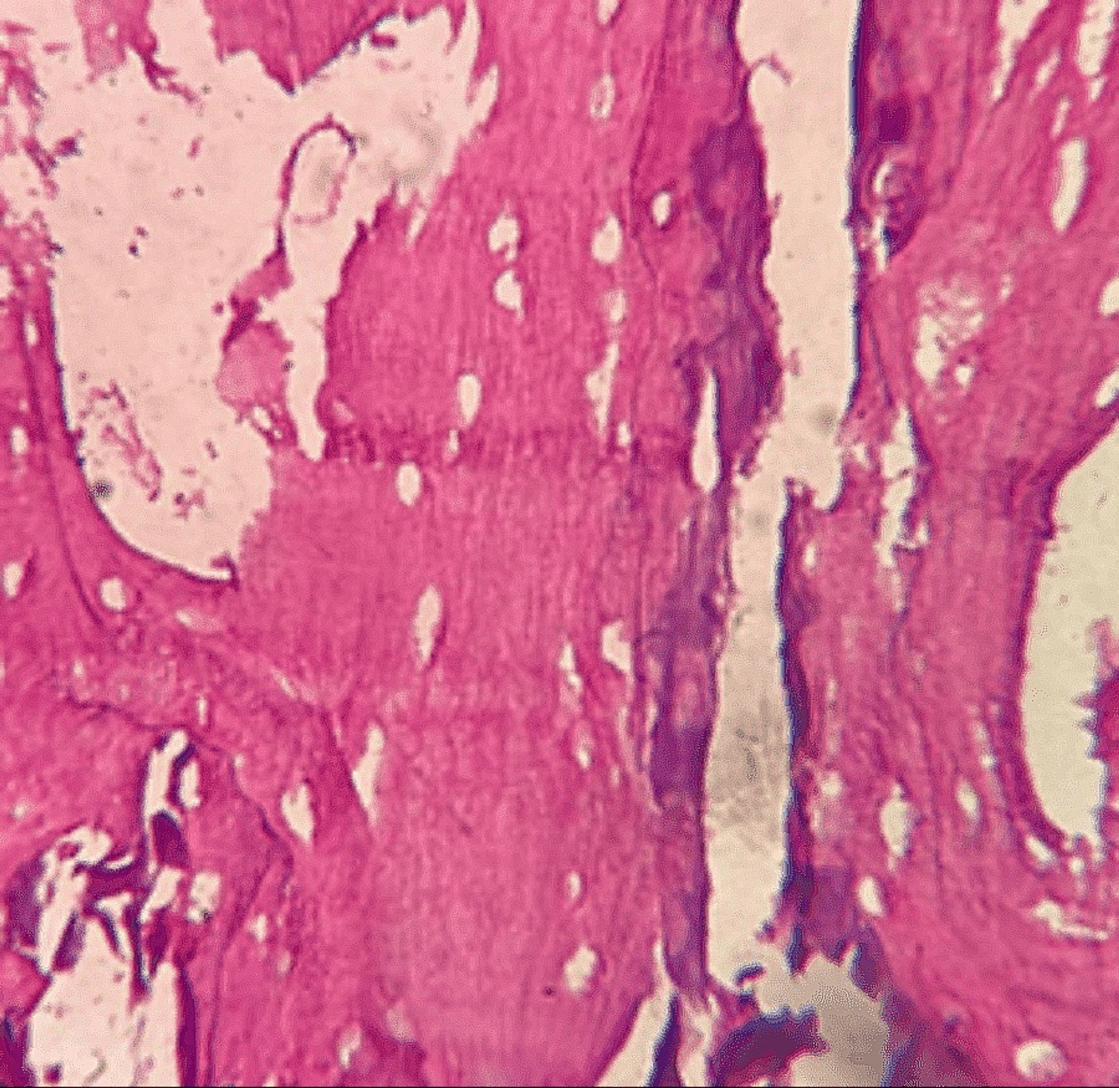
Group A DECM sample after H & E staining H & E: hematoxylin & eosin, DECM: decellularised extracellular matrix

**Figure 8:**
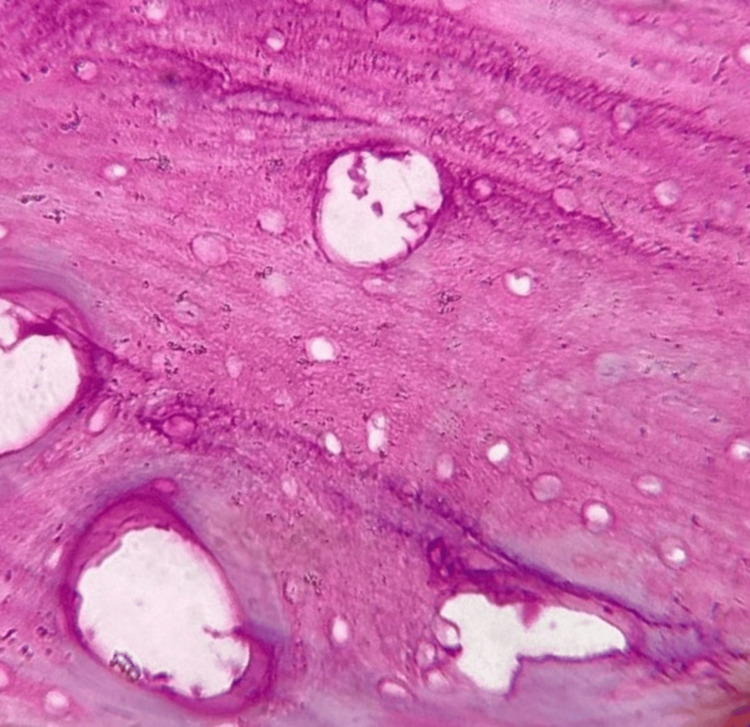
Group B DECM sample after H & E staining H & E: hematoxylin & eosin, DECM: decellularised extracellular matrix

**Figure 9:**
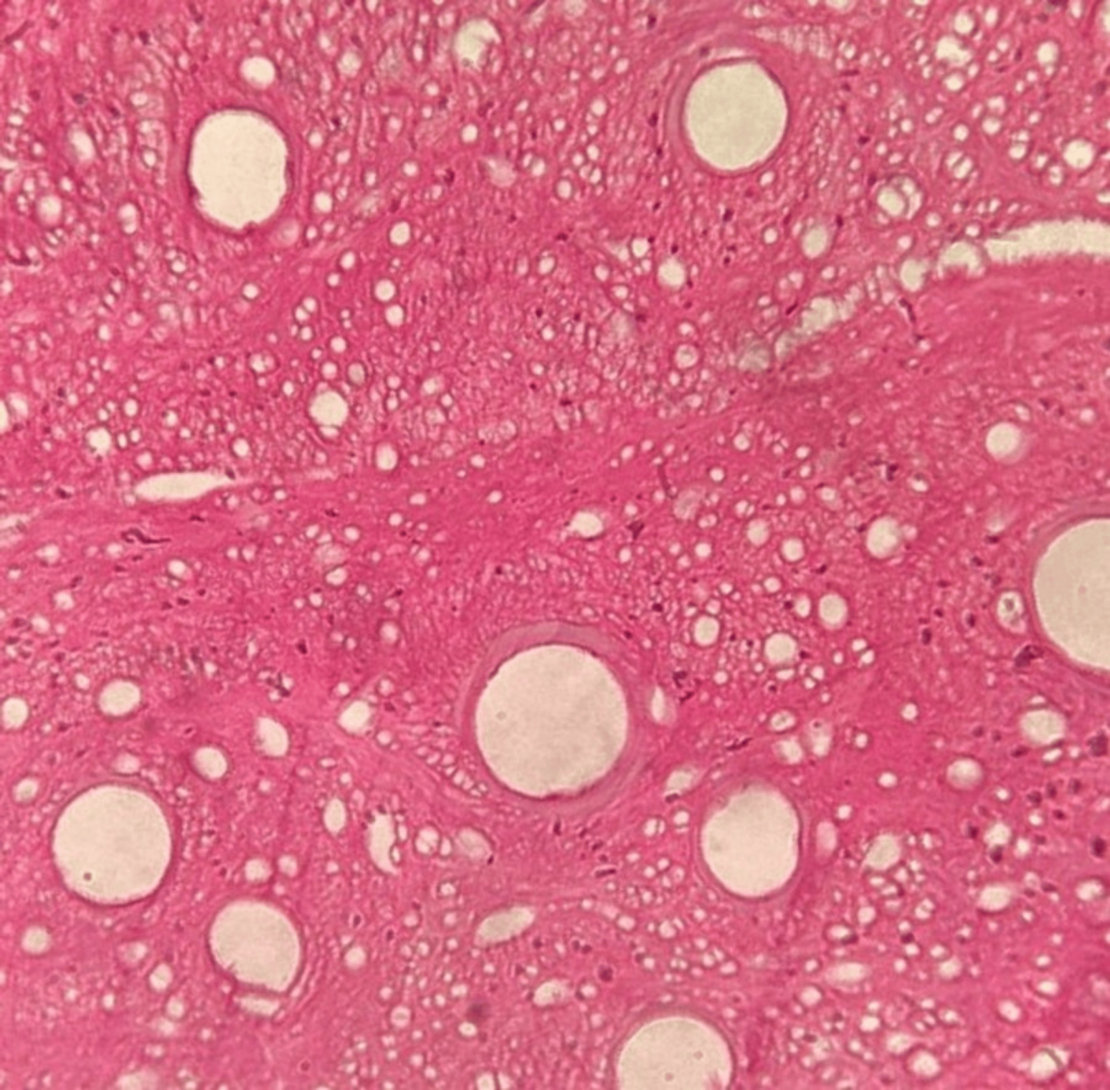
Group C DECM sample after H & E staining H & E: hematoxylin & eosin, DECM: decellularised extracellular matrix

On H & E analysis, the sections of samples from all three groups when viewed under the microscope suggested of decellularised ECM of bone, as observed in Figures 7-9. All three groups revealed adequately decellularised structures with empty lacunae spaces devoid of cellular and nuclear material. The lamellar arrangement of the ECM structure was well appreciable in all the groups. However, some degree of damage to the ECM fibers was noted with Group A. The ECM structure appeared intact with limited damage to the fibers in Group B and Group C. When decellularized collagen tissues were treated with Triton X-100, sodium deoxycholate (SDC), sodium dodecyl sulfate (SDS), and 3-[(3-cholamidopropyl) dimethylammonio]-1-propanesulfonate (CHAPS), comparable outcomes were seen [17]. This proves that our developed protocols of Group B and C were comparable to the protocols performed using common decellularising chemical agents. 

Biocompatibility analysis

**Figure 10:**
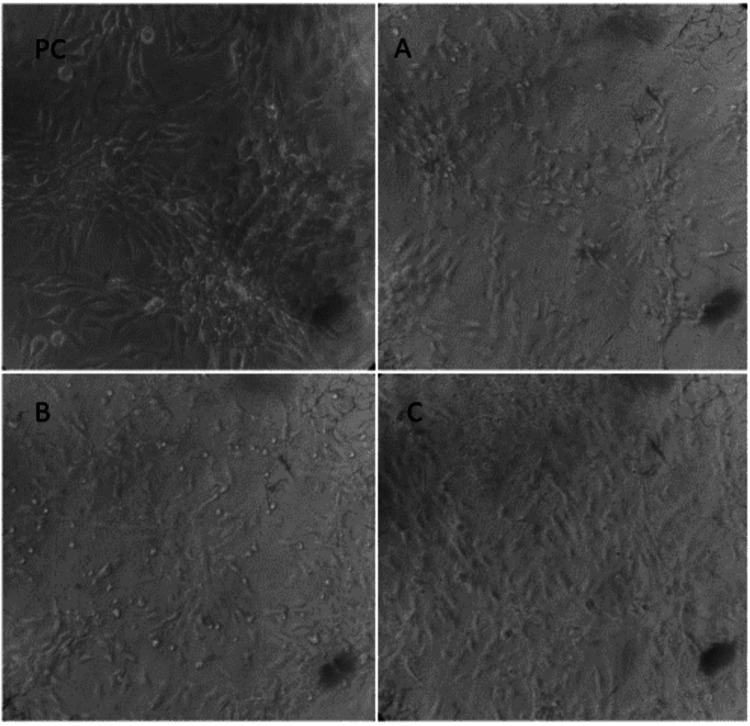
Confocal analysis showing good cell viability PC: positive control, A: Group A, B: Group B, C: Group C

**Figure 11:**
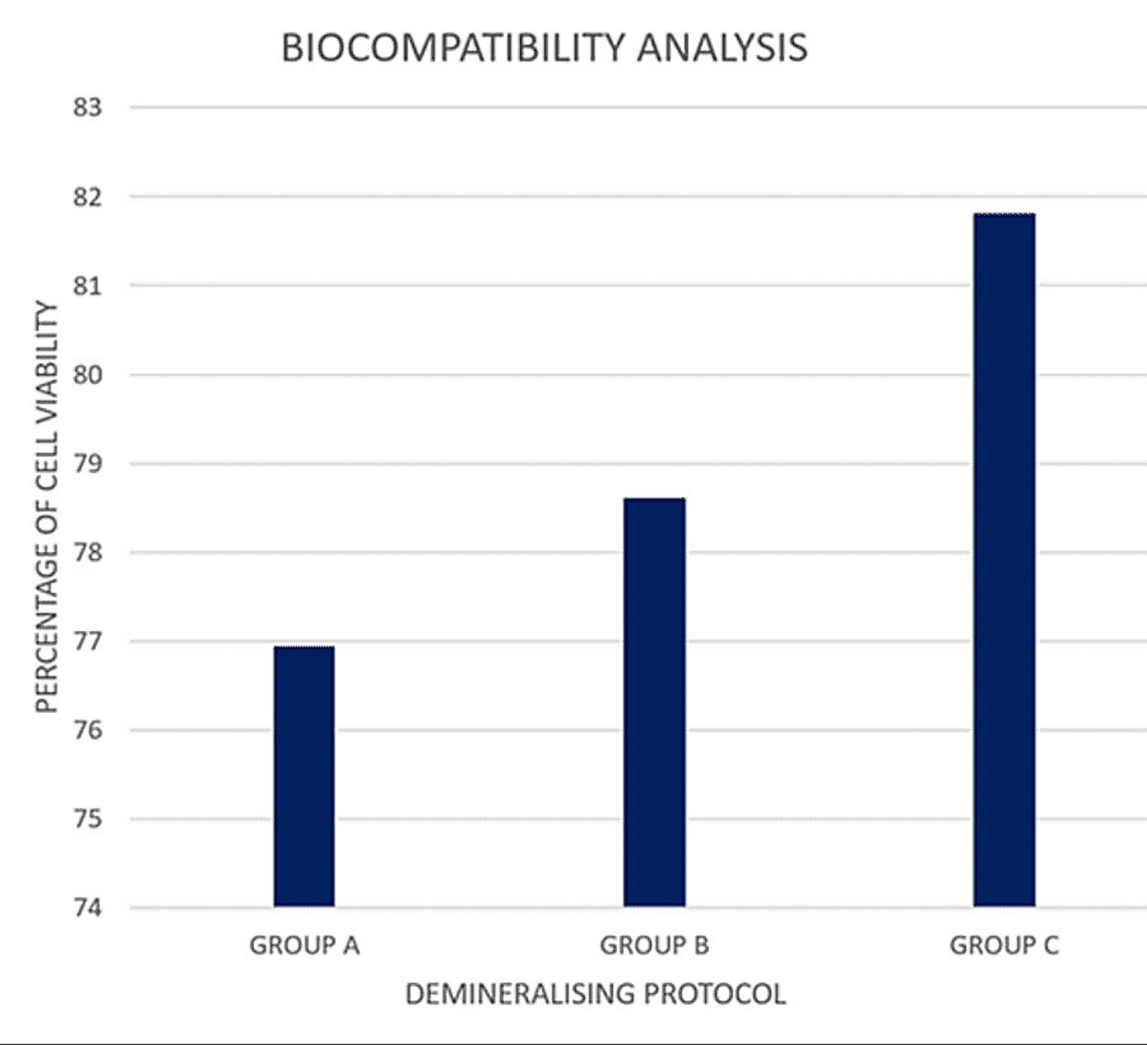
Effect of the three protocols on the biocompatibility of the DECM samples DECM: decellularised extracellular matrix

On MTT analysis, all three groups showed viable cells that maintained their morphological characteristics, with Group C revealing the highest percentage of cell viability and morphologic similarity to the positive control. The results have been depicted in Figures 10, 11 and Table 1. All three groups revealed a statistically significant difference in cell viability in comparison with the negative control. On comparison of the three groups, no statistically significant difference was observed between the groups with respect to the percentage of cell viability. The results show that all three groups are biocompatible and can be further explored for bioprinting and tissue engineering applications.

**Table 1:** Effect of the three protocols on the biocompatibility of the DECM samples DECM: decellularised extracellular matrix

PROTOCOL	N	PERCENTAGE OF CELL VIABILITY	STANDARD DEVIATION	P VALUE
GROUP A	3	76.94	3.576	0.940
GROUP B	3	78.61	5.766	0.705
GROUP C	3	81.81	4.622	0.389

## Discussion

Treatment of infrabony defects with predictable regeneration of bone has remained a challenge over the years. With the advances in tissue engineering and regenerative medicine, 3D bioprinted scaffolds have been employed in the treatment of bony defects. Though various biomaterials and cells have been used, the application of ECM-based therapeutics has gained attention due to their advantages. The unique and specialised tissue-specific ECM when applied along with 3D bioprinting technologies, recreates the inherent microenvironmental niche in the 3D printed constructs. 

Each tissue has its unique ECM components organised in a distinct pattern specific to the form and function of the tissue [18]. Majorly ECM components can be broadly categorised as fibrous proteins, glycoproteins, signaling molecules, and matrix receptors [18,19]. Collagen and elastin are the predominant fibrillar proteins that exhibit robust mechanical strength. The synthesis of collagen is a highly coordinated and conserved process that is controlled by various auto and paracrine factors. Glycoproteins form the core of the ECM and provide support for the ECM components. The hydrodynamic properties of proteoglycans and glycosaminoglycans provide turgidity and compressibility that give unique structural and mechanical strength to ECM. Adhesive proteins like fibronectin, laminin integrin, and nidogen enable cell interactions during wound healing, repair, and regeneration [20]. Growth and signaling factors expressed by the ECM create a niche of biologic cues that modulate cell behavior [21]. They also act as a reservoir of cryptic bioactive signaling peptides that play a significant role during tissue regeneration [22]. 

Though well proven, the various features of the native ECM that are essential for tissue regeneration cannot be replicated solely by an individual or even combinations of single ECM-based proteins [23]. In contrast, DECM acts as a natural storehouse for many bioactive signaling chemicals. By retaining most of the native ECM's structural and functional elements, these molecules continue to operate without inducing an immune reaction [24]. This can be achieved by the process of decellularisation, which removes all the cellular and nuclear matter that is responsible for the immune and inflammatory host response. Studies have shown efficiently decellularised allogenic and xenogenic tissue constructs that can be used as potential alternatives to synthetic and single ECM-based biomaterials [9,25]. 

The optimal decellularisation protocol is a tissue-dependent procedure [26]. With bone tissue being a unique connective tissue composed of the mineral matrix, achieving optimum decellularisaation of the bone ECM also requires demineralisation of the mineral housing. Hence, for bone tissue, a synergistic approach of both demineralisation and decellularization needs to be applied. Conventionally, various demineralisation and decellularisation protocols can be categorised broadly as physical, chemical, enzymatic, and combination approaches [12]. Physical methods include freeze-thaw cycles, immersion-agitation, sonication, osmosis. Snap freezing leads to cell lysis caused by intracellular crystalisation. The cell remnants are then removed following physical agitations. Mechanical abrasions and osmosis allow cell dissociation from the underlying basement membrane. The application of electric micropulse destabilises the membrane potentials causing micropore formation and leading to decellularisation. However, physical methods alone are insufficient for complete decellularisation and adjunctive chemical or enzymatic processing is further required [27].

Chemical treatment with acid-bases, ionic-nonionic, and hypotonic-hypertonic solutions have been effective in decellularisation processes. Acetic acid, sulfuric acid, hydrochloric acid, and alkali agents like sodium sulfide, calcium hydroxide, sodium hydroxide, and ammonium hydroxide remove cellular and nuclear material by hydrolytic degeneration. Ionic and zwitterionic detergents like SDS, SDC, and Triton X 200 remove the immunologic material by membrane solubilisation and protein dissociation [28]. Hypertonic and hypotonic treatment with Tris HCl and NaCl induces osmotic imbalance resulting in cell lysis. 

Enzymatic treatments are a more specific form of decellularisation protocol. Nucleases, proteases, collagenases, trypsin, pepsin, lipases, and calcium chelating agents have been applied for effective cleaving of proteins and cellular bonds, hydrolysis of bonds of deoxyribonucleotide and ribonucleotide chains. Although enzymatic treatments are highly specific, achieving complete and effective removal of immunogenic components with enzymes alone is difficult. Some amount of enzymatic remnants post-treatment may hamper the cell adhesion and growth.

Currently, there is no gold standard protocol for the effective demineralisation and decellularisation process of the bone [12,26]. The present study analysed three different demineralisation protocols to best identify the optimal protocol for the decellularisation of bone samples. The samples were analysed for surface morphology, elemental composition, microanatomy, and biocompatibility post-decellularisation processing. 

On SEM analysis, both Group C and Group B revealed a porous architecture with well-connected and patent pores. Group C had a greater average pore size of 218.87µm in comparison with the average pore size of Group B which was 34.62µm. This difference in the pore size may be attributed to the variation in the inherent anatomy of the bone samples. Both Groups B and C have a pore size that is conducive to osteoblast migration and proliferation. The porosities in Group C were well appreciable in comparison to Group B which may be attributed to the improved demineralising effect of the Group C protocol. The results were in accordance with recent studies analysing the decellularisation of porcine annulus fibrous scaffold using Triton X-100, SDS, and trypsin [15]. This proves that the decellularisation protocols followed in this study had a similar effect on the surface properties of the tissues as the commonly used decellularisation protocols. 

On comparative analysis of the elemental composition of the decellularised samples, all three groups suggested the presence of ECM component collagen which is represented by the peaks of carbon and oxygen. All the protocols showed very little or no traces of calcium that prove the optimal demineralisation of the bone samples. Group A and Group B show the presence of trace amounts of nitrogen that may be attributed to the presence of proteins. These results prove that the three different protocols used in this study for demineralisation and decellularisation of the bone samples were effective in the procurement of DECM of bone.

H & E staining revealed optimum decellularisation observed with all three groups with the removal of cellular and nuclear components. Empty lacunae spaces were well appreciable. The lamellar structure of the DECM was observed in all three groups however, some amount of damage to the structure was noted in Group A. The damage to the ECM structure may be because of the exposure of the tissue to acidic agents along with physical agitation for a longer period of time. As retaining the structural and biochemical integrity of the ECM is of priority, following a less rigorous protocol for a short period of time would be more appropriate. Similar results were observed in a study that used different decellularisation protocols on mouse ovaries which showed less dense structures with remaining cell nuclei not visible [29]. 

All three groups showed optimum cell viability on MTT analysis with a statistically significant difference noted in relation to the negative control, proving that they are biocompatible for bioprinting applications. Group C revealed the highest percentage of cell viability and morphologic similarity to the positive control. These results are further corroborated by another study which stated that decellularised tissues using SDS, SDC, CHAPS, and Triton X-100 are devoid of harmful components and residual detergents at levels that are toxic to the cells [30]. They concluded that in relation to cell viability, Triton X-100 had shown the greatest cell viability in contrast to SDS, SDC, and CHAPS.

On consideration of all the different aspects analysed, the decellularised samples of Group C were found to be suitable for further bioink preparation. They revealed the best surface characteristics on SEM analysis, with fibrillar structure and well-interconnected porosities. They also had a higher carbon and oxygen content on EDX analysis which may be attributed to the presence of collagen. On correlating H & E staining, and biocompatibility analysis, Group C showed optimum decellularisation and biocompatibility that would be acceptable for further bioink preparation for tissue engineering applications. This proves that the protocol followed in Group C was optimum for effective demineralisation and decellularisation while maintaining the essence of the structural, functional, and compositional elements of the native ECM matrix. 

However, it is important to note that the results of this study are too preliminary and cannot be extrapolated to clinical success, which remains the limitation of this study. Further, biochemical analysis, cell culture analysis and animal studies to assess osteogenic potential and genetic marker expression of the DECM must be conducted to substantiate the results of this study. The biochemical analysis will provide us with crucial information on the effect of the demineralisation and decellularisation protocols on the key tissue components like collagen, remanent DNA, sulfated glycosaminoglycan levels etc. Cell culture analysis would also add on to strengthen the results of this study by providing information on cell viability and proliferation rate. In vitro tests should focus on the application of the developed DECM as a bioink formulation by optimising the rheological, printing, and biologic properties of the bioink, further paving the way for application of DECM-based 3D bioprinted constructs in animal models. 

## Conclusions

In conclusion, variations in DECM properties are particularly important while considering their application as a bioink component with highly sensitive or delicate cell types, such as stem cells or progenitor cells. Therefore, identifying an optimized protocol is crucial for developing DECM-based bioinks for 3D bioprinting applications.

The different demineralisation and decellularisation protocols examined in this study appear promising, with the protocol used by Group C demonstrating the most potential for DECM-based bioink applications. Further research is needed to evaluate the suitability of the obtained DECM following Group C protocol, for creating tissue-specific bioinks for 3D bioprinting. Conversion of this DECM into bioink formulation along with other ossifying agents like hydroxyapatite and beta-tricalcium phosphate grafts would allow bioprinting of 3D osseous scaffolds, that can be used for the treatment of infrabony defects that are often seen in periodontal disease. These constructs would not only act as scaffolds for cell attachment and proliferation but also provide crucial biomimetic cues for better regenerative outcomes. It would provide a more personalised and precise bone grafting option for the treatment of bony defects. 
